# Discovery of CD8 T cell epitopes in Merkel cell polyomavirus through combinatorial encoding with MHC multimers

**DOI:** 10.1186/2051-1426-1-S1-P13

**Published:** 2013-11-07

**Authors:** Rikke Lyngaa, Natasja W Pedersen, David Schrama, Charlotte A Thrue, Özcan Met, Dafina Ibrani, Per thor Straten, Paul Nghiem, Jürgen C Becker, Sine R Hadrup

**Affiliations:** 1 Center for Cancer Immune Therapy (CCIT), Department of Hematology, University Hospital Herlev, Copenhagen, Denmark; 2 General Dermatology, Medical University of Graz, Graz, Austria; 3 Departments of Medicine/Dermatology, Pathology, University of Washington, Fred Hutchinson Cancer Research Center, Seattle, WA, USA

## 

Merkel cell carcinoma (MCC) is a highly aggressive skin cancer induced through oncogenic transformation by the Merkel cell polyomavirus (MCPyV). Intratumoral CD8 T cell infiltration and the immunogenic capacity of the host have been strongly correlated to survival in patients with MCC. The MCPyV oncoproteins represent highly attractive targets for immunotherapy of MCC, being both foreign to the host and specific for the infection. We have mapped the T cell recognition of MCPyV by use of a novel technology platform for T-cell enrichment and combinatorial encoding of histocompatibility antigen (HLA) class I multimers. We investigated the T cell recognition of both the oncogenic proteins, Large and Small T antigen and the capsid protein VP1 that is profoundly expressed also in the non-symptomatic MCPyV infected healthy individuals. We investigated the T cell recognition of 398 in silico predicted HLA-ligands from Large T, Small T and VP1 and identified T-cell responses restricted to HLA-A1, A2, A3, A11 and B7. Numerous T cell responses were detected against MCPyV in both healthy donors and MCC patients, with a striking observation that T cell responses against Large and Small T antigens were found exclusively in MCC patients, suggesting that these oncoproteins are only presented to the immune system in the tumor-bearing host and represent highly specific targets for cancer immunotherapy of MCC. Importantly, we show for three different epitopes that MCC specific T cells are capable of killing target cells expressing Large T antigen, including HLA-matched MCC cell lines, indicating that these T cells could provide functional activity upon in vivo transfer, and thus be employed in adoptive T cell therapy for MCC. T cell epitopes from Large T antigen of MCPyV display a large sequence variation with other polyomaviruses, but are highly conserved among different strains of MCPyV. Targeting these epitopes are therefore likely to lead to specific recognition of the tumor cells bearing the Large T integration - that is essential for growth and survival of the tumor cells.

